# Nutrients and Inflammatory Diseases

**DOI:** 10.1155/2017/6134909

**Published:** 2017-04-28

**Authors:** Jie Yin, Michael Conlon, Sung Woo Kim

**Affiliations:** ^1^Key Laboratory of Agro-Ecological Processes in Subtropical Region, Institute of Subtropical Agriculture, Chinese Academy of Sciences, Scientific Observing and Experimental Station of Animal Nutrition and Feed Science in South-Central, Ministry of Agriculture, Hunan Provincial Engineering Research Center for Healthy Livestock and Poultry Production, Changsha, Hunan 410125, China; ^2^University of Chinese Academy of Sciences, Beijing 100039, China; ^3^CSIRO Food and Nutrition, Canberra, ACT, Australia; ^4^North Carolina State University, Raleigh, NC, USA

Inflammation has been widely demonstrated to be involved in various stimuli, such as oxidative stress, bacterial and virus infection, and some physiological process, while a chronic and excessive inflammatory response is a significant risk factor for developing various human diseases [1].

An increasing number of compelling reports are recently published suggesting that some nutrients, like amino acids, oligosaccharides, and short-chain fatty acids, exhibit anti-inflammatory effect [2, 3], which will help the understanding of nutritional contributions in the treatment and control of certain inflammatory diseases. Meanwhile, nutrients show a close relationship with the gut microbiota, which further influences gastrointestinal inflammatory responses [3]. Little is known about how this relationship is affected by dietary nutrients that alleviate inflammation via mediating the composition and richness of the microbiota. Also, molecular mechanisms of selected nutrients functioning to alleviate inflammatory diseases have not been clearly investigated. Thus, there is an urgent need to advance scientific knowledge on nutrients alleviating inflammatory diseases.

The articles contained in this special issue include 4 review papers and 10 original research papers that are focused on characterizing the contribution and molecular mechanisms associated with nutrients and inflammation. A brief description of these 14 works is detailed below.

Amino acids and their metabolites have been widely demonstrated to exhibit anti-inflammatory effect on various inflammatory models, such as inflammatory bowel disease. X. Bao et al. provide a detailed review of the literature on the relationship between amino acids and inflammatory bowel disease in their paper titled “Roles of Dietary Amino Acids and Their Metabolites in Pathogenesis of Inflammatory Bowel Disease.”

In the paper titled “Role of Uric Acid Metabolism-Related Inflammation in the Pathogenesis of Metabolic Syndrome Components Such as Atherosclerosis and Nonalcoholic Steatohepatitis,” A. Kushiyama et al. outline the molecular mechanisms underlying inflammation occurrence in relation to uric acid metabolism.

Inflammation contributes to the development of various metabolic diseases, such as obesity and diabetes. S. Chen et al. review the anti-inflammatory properties of quercetin in relation to obesity and type 2 diabetes in the paper titled “Therapeutic Effects of Quercetin on Inflammation, Obesity, and Type 2 Diabetes.”

Intestinal microbiota is highly involved in host physiology and pathology through activity of the microbiome and its metabolic products. “Diet-Intestinal Microbiota Axis in Osteoarthritis: A Possible Role” by Y. Li et al. concludes that intestinal microbiota is a major hidden risk factor for osteoarthritis and an important explanation for person-level risk factors.

Four groups from R. Garib et al., H. Xiao et al., H. Ni et al., and M. Li et al. investigated the anti-inflammatory effects of amino acids on different inflammatory models. The papers include “Effect of Previous High Glutamine Infusion on Inflammatory Mediators and Mortality in an Acute Pancreatitis Model,” “N-Acetyl-L-cysteine Protects the Enterocyte against Oxidative Damage by Modulation of Mitochondrial Function,” “Effects of Glutamate and Aspartate on Serum Antioxidative Enzyme, Sex Hormones, and Genital Inflammation in Boars Challenged with Hydrogen Peroxide,” and “High-Methionine Diet Attenuates Severity of Arthritis and Modulates IGF-I Related Gene Expressions in an Adjuvant Arthritis Rats Model.”

In the article “In Vitro Anti-Inflammatory Effects of Three Fatty Acids from Royal Jelly,” Y.-F. Chen et al. evaluate and compare the in vitro anti-inflammatory effects of three fatty acids on lipopolysaccharide-stimulated RAW 264.7 macrophages and find that MAPK and NF-*κ*B signaling pathways involve in the mechanism of anti-inflammatory effects of fatty acids from royal jelly.

In rats infected with enterotoxigenic *Escherichia coli*, G. Liu et al. find that dietary chitosan markedly alleviated intestinal inflammation. The paper is titled “Chitosan Modulates Inflammatory Responses in Rats Infected with Enterotoxigenic *Escherichia coli*.”

Traditional medical plants and plant extracts have been widely explored to treat inflammatory diseases. In this issue, two articles about curcumin and *Veronicastrum axillare* were collected: “Curcumin Alters Neural Plasticity and Viability of Intact Hippocampal Circuits and Attenuates Behavioral Despair and COX-2 Expression in Chronically Stressed Rats” by G.-Y. Choi et al. and “*Veronicastrum axillare* Alleviates Lipopolysaccharide-Induced Acute Lung Injury via Suppression of Proinflammatory Mediators and Downregulation of the NF-*κ*B Signaling Pathway” by Q. Ma et al.

A clinical trial titled “The Effect of Immunonutrition on the Postoperative Complications in Thymoma with Myasthenia Gravis” by Y. Xin et al. concludes that preoperative immunonutrition support is effective in reducing postoperative complications in patients of thymoma with myasthenia gravis.

High-mobility group box 1 protein (HMGB1) and autophagy are vital to maintain cellular homeostasis and protect against inflammatory response. In the paper titled “Regulation of Autophagy-Related Protein and Cell Differentiation by High Mobility Group Box 1 Protein in Adipocytes” by H. Feng et al., it focuses on the relationship between HMGB1 and autophagy in adipocytes.

## References

[1] Nasef N. A., Mehta S., Ferguson L. R. (2017). Susceptibility to chronic inflammation: an update. *Archives of Toxicology*.

[2] Da Silva M. S., Rudkowska I. (2015). Dairy nutrients and their effect on inflammatory profile in molecular studies. *Molecular Nutrition & Food Research*.

[3] Velloso L. A., Folli F., Saad M. J. (2015). TLR4 at the crossroads of nutrients, gut microbiota, and metabolic inflammation. *Endocrine Reviews*.

